# Timing Is Everything: Impact of Naturally Occurring *Staphylococcus aureus* AgrC Cytoplasmic Domain Adaptive Mutations on Autoinduction

**DOI:** 10.1128/JB.00409-19

**Published:** 2019-09-20

**Authors:** Tim J. Sloan, Ewan Murray, Maho Yokoyama, Ruth C. Massey, Weng C. Chan, Boyan B. Bonev, Paul Williams

**Affiliations:** a Centre for Biomolecular Sciences, School of Life Sciences, University of Nottingham, Nottingham, United Kingdom; b School of Cellular and Molecular Medicine, University of Bristol, Bristol, United Kingdom; c School of Pharmacy, Centre for Biomolecular Sciences, University of Nottingham, Nottingham, United Kingdom; University of Illinois at Chicago

**Keywords:** AgrC, *Staphylococcus aureus*, accessory gene regulator (*agr*), autoinducing peptide (AIP), exotoxins, molecular dynamics, quorum sensing, sensor kinase, virulence

## Abstract

Mutations in the polymorphic Staphylococcus aureus *agr* locus responsible for quorum sensing (QS)-dependent virulence gene regulation occur frequently during host adaptation. In two genomically closely related S. aureus clinical isolates exhibiting marked differences in Panton-Valentine leukocidin production, a mutation conferring an N267I substitution was identified in the cytoplasmic domain of the QS sensor kinase, AgrC. This natural mutation delayed the onset and accumulation of autoinducing peptide (AIP) and showed reduced responsiveness to exogenous AIPs. Other S. aureus strains harboring naturally occurring AgrC cytoplasmic domain mutations were identified, including T247I, I311T, A343T, L245S, and F264C. These mutations were associated with reduced cytotoxicity, delayed/reduced AIP production, and impaired sensitivity to exogenous AIP. Molecular dynamics simulations were used to model the AgrC cytoplasmic domain conformational changes arising. Although mutations were localized in different parts of the C-terminal domain, their impact on molecular structure was manifested by twisting of the leading helical hairpin α1-α2, accompanied by repositioning of the H-box and G-box, along with closure of the flexible loop connecting the two and occlusion of the ATP-binding site. Such conformational rearrangements of key functional subdomains in these mutants highlight the cooperative response of molecular structure involving dimerization and ATP binding and phosphorylation, as well as the binding site for the downstream response element AgrA. These appear to increase the threshold for *agr* activation via AIP-dependent autoinduction, thus reducing virulence and maintaining S. aureus in an *agr*-downregulated “colonization” mode.

**IMPORTANCE** Virulence factor expression in Staphylococcus aureus is regulated via autoinducing peptide (AIP)-dependent activation of the sensor kinase AgrC, which forms an integral part of the *agr* quorum sensing system. In response to bound AIP, the cytoplasmic domain of AgrC (AgrC-cyt) undergoes conformational changes resulting in dimerization, autophosphorylation, and phosphotransfer to the response regulator AgrA. Naturally occurring mutations in AgrC-cyt are consistent with repositioning of key functional domains, impairing dimerization and restricting access to the ATP-binding pocket. Strains harboring specific AgrC-cyt mutations exhibit reduced AIP autoinduction efficiency and a timing-dependent attenuation of cytotoxicity which may confer a survival advantage during established infection by promoting colonization while restricting unnecessary overproduction of exotoxins.

## Abstract (precis)

Virulence factor expression in Staphylococcus aureus is regulated via autoinducing peptide (AIP)-dependent activation of the sensor kinase AgrC, which forms an integral part of the *agr* quorum sensing system. In response to bound AIP, the cytoplasmic domain of AgrC (AgrC-cyt) undergoes conformational changes resulting in dimerization, autophosphorylation, and phosphotransfer to the response regulator AgrA. Naturally occurring mutations in AgrC-cyt are consistent with repositioning of key functional domains, impairing dimerization and restricting access to the ATP-binding pocket. Strains harboring specific AgrC-cyt mutations exhibit reduced AIP autoinduction efficiency and a timing-dependent attenuation of cytotoxicity which may confer a survival advantage during established infection by promoting colonization while restricting unnecessary overproduction of exotoxins.

## INTRODUCTION

Virulence gene regulation in Staphylococcus aureus relies on a finely balanced network of transcriptional and translational regulators. These systems integrate the response to a wide variety of stimuli, including pH, oxidative stress, temperature, cell wall damage, nutrient availability, and cell population density (1–3). With respect to the latter, the accessory gene regulator (*agr*) is a cell-cell communication or “quorum sensing” (QS) system integral to the staphylococcal virulence factor regulatory network. It responds to bacterial cell population density by sensing a threshold concentration of the cognate QS signal molecule. This leads to the upregulation of diverse exotoxin genes (including Panton-Valentine leukocidin [PVL] and α-toxin) while downregulating cell wall proteins responsible for, e.g., surface attachment and biofilm formation (1, 2). As such, it is understood to play a crucial role in the transition from host colonization to invasion.

While *agr* activation *in vitro* occurs during the late exponential/early stationary phases of growth, the situation *in vivo* appears to be much more complex with both spatial and temporal factors at work (1–3). Confined environments accelerate accumulation of the cognate QS signal molecule (4), while exposure to environmental stressors modulate the threshold for *agr* activation through other regulatory systems (5). Studying this virulence regulatory network holds promise for understanding the factors contributing to infection severity and aiding the design of “antivirulence” agents that control infection by inhibiting virulence factor production rather than bacterial growth (6, 7).

**FIG 1:**
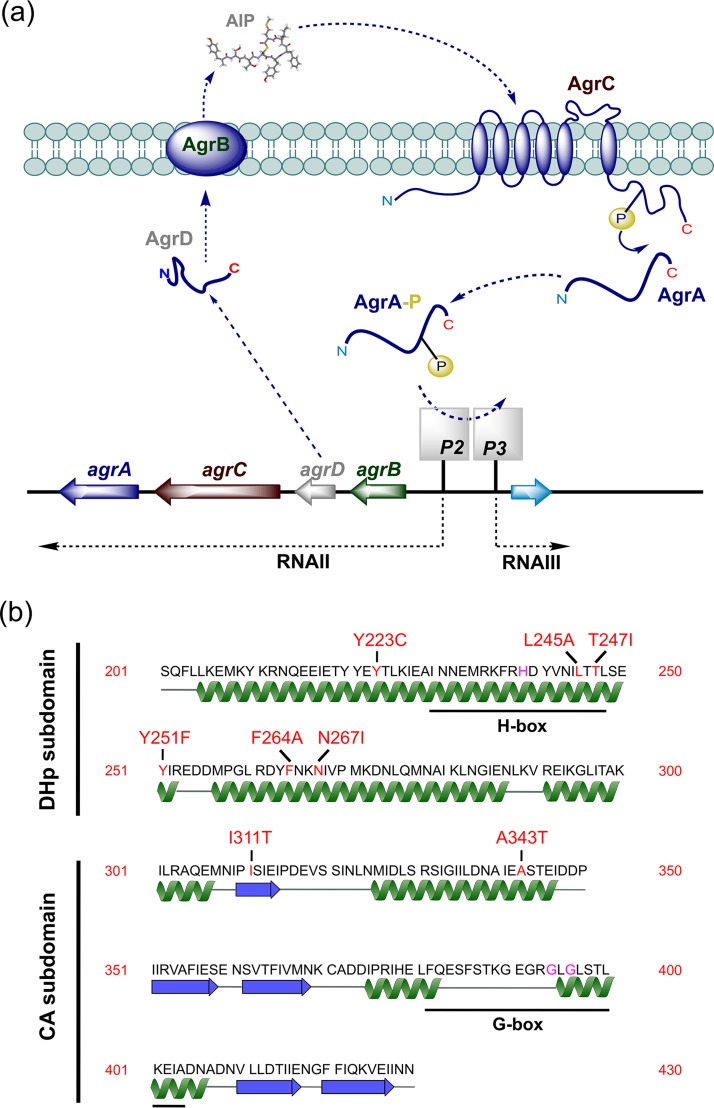
(a) Schematic of the *agr*-dependent quorum-sensing system. Two divergent transcripts (RNAII and RNAIII) are driven by the *agr*P2 and *agr*P3 promoters, respectively. The *agrBDCA* operon is in RNAII. The autoinducing peptide (AIP) is generated from the AgrD peptide precursor via AgrB. The sensor kinase, AgrC, autophosphorylates on binding AIP, resulting in phosphorylation of the response regulator AgrA to activate the P2 and P3 promoters. Downstream target genes are activated by AgrA either directly or indirectly via RNAIII, which also codes for delta-hemolysin. (b) Secondary structure of the cytoplasmic domain of AgrC indicating the location of the dimerization and histidine phosphorylation (DHp) and catalytic and ATP-binding (CA) subdomains. The key functional and mutated residues are marked in purple and red, respectively. The H-box histidine (His239) is the predicted phosphorylation site, and G-box glycine residues (Gly394 and Gly396) are critical for ATP binding. Functional annotations were adapted from Cisar et al. (37).

The *agr* locus consists of two divergent transcriptional units (*agrBCDA* and RNAIII), controlled by the *agr*P2 and *agr*P3 promoters, respectively (8). AgrA and AgrC constitute a two-component system (TCS) in which the transmembrane AgrC is the sensor kinase and cytoplasmic AgrA is the response regulator (Fig. 1a). AgrC is activated by binding an autoinducing peptide (AIP) and phosphorylates AgrA, which binds to the P2 promoter upregulating *agrBCDA*, conferring a positive-feedback loop that autoinduces AIP production and so drives virulence factor production directly via AgrA or via the AgrA-dependent P3 operon (8).

AgrC, which functions as a dimer, has a modular architecture consisting of a transmembrane N-terminal sensory module incorporating an AIP-binding site linked to a C-terminal cytoplasmic module that contains two subdomains (8). These are the dimerization and histidine phosphorylation (DHp) and catalytic and ATP-binding (CA) subdomains (8), which incorporate the H box that is phosphorylated via the G-box kinase (Fig. 1b).

AgrC appears to behave as a rheostat, where activation on AIP-binding results in the twisting of a helical linker relative to the cytoplasmic domain and subsequent dimerization that results in AgrA phosphorylation (9). Recently, Xie et al. (10) identified a key noncovalent interaction between R238 and Q305 that stabilizes AgrC in the “off” state. This “latch” is proposed to lift following the binding of AIP to the extracellular AgrC sensor domain, thus mediating the structural changes that result in AgrC activation. Consequently, replacement of R238 with A renders AgrC constitutively active (10).

The divergent nature of the S. aureus *agr* locus particularly with respect to the region containing *agrB*, *agrD*, and *agrC* in part reflects the structural variations in the four AIPs that are accompanied by compensatory changes in *agrB* and *agrC* (1, 11). For subgroups of the latter, these predominantly localize to the sensory module in contrast to the AgrC cytoplasmic domain, which is highly conserved. For example, the cognate AIPs for the AgrC1 and AgrC4 sensor kinases, AIP-1 and AIP-4, respectively, differ by a single amino acid. Differential recognition of AIP-1 by AgrC4 depends on only three amino acid changes in the AgrC4 extracellular loop 2 (11). However, the frameshifts, insertions, deletions, and substitutions in *agr* genes, primarily *agrA* and *agrC*, that arise during host adaptation (12–15) generally result in complete *agr* inactivation. For example, Mairpady Shambat et al. (15) isolated a methicillin-resistant S. aureus (MRSA) ST22 lineage strain harboring a single AgrC Y223C cytoplasmic domain substitution (Fig. 1b) that switched the virulence phenotype from cytotoxic to colonizing but was reversed on mutating back to C223Y. However, not all naturally occurring *agr* mutations are likely to be inactivating but may modify the timing and strength of *agr* induction. Here, we explore the experimental impact of naturally occurring AgrC cytoplasmic domain substitutions on exotoxin (PVL) and AIP production, focusing on the dynamics of *agr* activation and their impact on the conformation of the AgrC cytoplasmic domain using molecular dynamics simulations. The findings presented have potential implications for the adaptation of S. aureus during infection and the clinical potential of *agr* antagonists as antivirulence agents.

## RESULTS

### AIPs, *agr*, and PVL production and inhibition.

**FIG 2:**
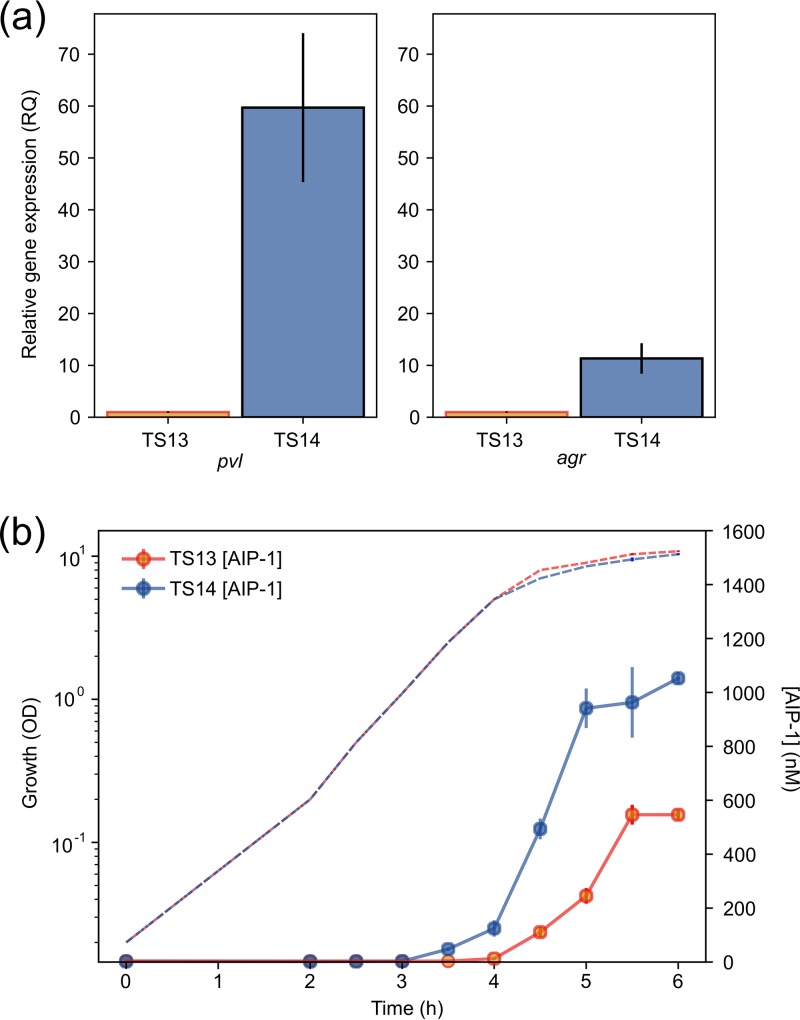
(a) Comparative expression of PVL (*lukF-PV*) and *agr* (RNAIII) in S. aureus strains TS13 and TS14; (b) AIP-1 production as a function of growth for TS13 and TS14. All assays were performed in triplicate, with error bars representing the standard errors of the mean (SEM). Hashed lines indicate the growth curves. RQ, relative quantification; OD, optical density.

**FIG 3:**
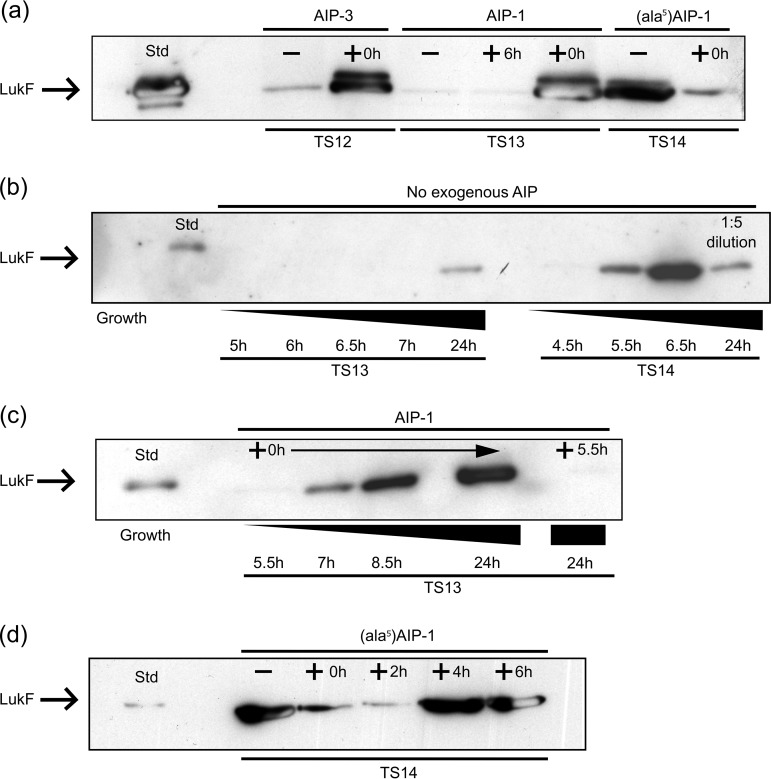
Western immunoblot analysis of PVL (LukF) production in S. aureus strains TS12 (*agr* group 3), TS13 (*agr* group I), and TS14 (*agr* group 1) in response to exogenous AIP. (a) PVL production in the absence of AIP (TS12, TS13, and TS14) or after the addition of AIP-1 (TS13) or AIP-3 (TS12) at the time of inoculation (time zero [0 h]; TS12 and TS13) or after 6 h (TS13); for TS14, (Ala^5^)AIP-1 was also added at 0 h. (b) LukF production profiles over time for TS13 and TS14. (c) PVL production profile over time after exogenous addition of AIP-1 to TS13 at 0 h or after 5.5 h. (d) PVL production by TS14 after 24 h is reduced when (Ala^5^)AIP-1 is added at 0 h or after 2 h but not after 4 or 6 h.

Among a group of S. aureus ST22 methicillin-sensitive (MSSA) strains isolated from clinical samples, we noted a subset of high-level PVL producers on immunoblotting, with one exception: strain TS13, a low-level PVL producer (16). To begin investigating the molecular basis for this difference, we compared TS13 with a high PVL producer, TS14. This strain, which had been isolated from a different patient at the same hospital 2 weeks earlier, shares the same *spa* type t005, and is closely related to TS13 at the genomic level (differing by only 193 single nucleotide polymorphisms [SNPs] [see Tables S3 and S4 in the supplemental material] while sharing 642 SNPs in common compared to an ST22 reference strain). To determine the relative expression levels of the PVL genes in TS13 and TS14, quantitative reverse transcription-PCR (RT-PCR) was carried out using *lukF-PV* and found to be 59.7-fold higher (95% confidence interval [CI] = 37.8 to 94.1, *P* < 0.01) in TS14 (Fig. 2a). Since differences in *agr* expression may account for the observed variation, we also examined *agr* transcription by measuring RNAIII levels and found an 11.3-fold difference (95% CI = 7.0 to 18.4, *P* < 0.05) between the two strains (Fig. 2a). This defect in PVL production in TS13 could be restored to TS14-like levels by provision of exogenous AIP-1 at the time of inoculation (0 h) (Fig. 3a and c). Similar results were also obtained with the low-level PVL producer, *agr* group 3 strain TS12, in response to exogenous AIP-3 (Fig. 3a). For TS14 but not TS13, PVL could be detected at the end of log phase approximately 5.5 h after inoculation (Fig. 3b). However, when AIP-1 was added to TS13 after 5.5 h growth, PVL was not restored (Fig. 3c). For TS14, PVL production was inhibited by the cross group *agr* inhibitor (Ala^5^)AIP-1 when added at 0 or 2 h after inoculation but not after 4 or 6 h of growth (Fig. 3d). These data confirm the *agr*-dependent nature of PVL production in these strains and show that there is a “window” period after which the exogenous cognate AIP or an AIP antagonist, respectively, is unable to activate or inhibit PVL production.

### Why does *agr* activation differ between TS13 and TS14?

Since PVL production in TS13 responds to exogenous AIP-1, we investigated whether the AIP production profiles differed between TS13 and TS14 as a function of growth phase. Cell-free culture supernatants were collected at intervals throughout growth and assayed using a bioluminescent AIP-1 bioreporter (11). Figure 2b shows that AIP-1 was detectable in TS14 supernatant ∼1 h before TS13, reaching a concentration of 1,052 ± 40 nM after 6 h compared to 546 ± 32 nM for TS13 (*P* < 1 × 10^−4^) (Fig. 2b). The differences in AIP-1 levels suggest that TS13 is unable to reach a threshold AIP concentration during an *agr*-responsive “window” period and thus unable to drive autoinduction of the QS circuitry. To determine whether mutations in *agr* and/or other PVL regulators could explain the TS13 PVL phenotype, the genome sequences of TS13 and TS14 were examined in more detail. In comparison to the reference AgrC1 protein from S. aureus NCTC8325 (GenBank accession no. CP000253.1), TS13 and TS14 contain a shared SNP resulting in a Y-to-F substitution at residue 251 (Y251F) (Fig. 1b). A SNP was also identified in the TS13 *agrC* gene, resulting in an N-to-I substitution at residue 267 (N267I) (Fig. 1b). An SNP conferring a D134Y substitution in SarU was also noted in TS13, but no other nonsynonymous mutations were identified in any of the other Sar proteins or major virulence regulators that impact *agr*, including Rot, MgrA, SaeRS, and SigB (1–3). No SNPs were identified in the *lukF-PV* genes that code for PVL toxins of either isolate. A list of all the SNPs varying between strains TS13 and TS14 is shown in the supplemental material.

**FIG 4:**
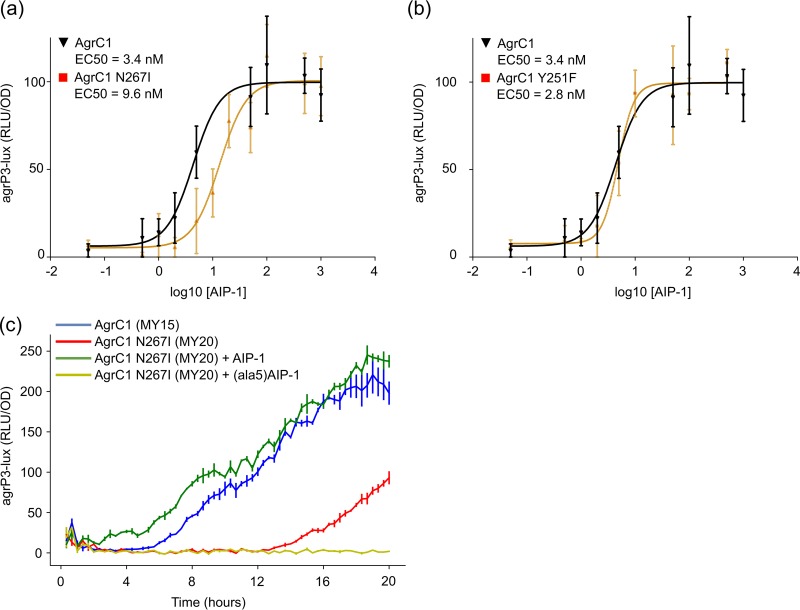
(a and b) Dose-response curves for the activation of the *lux*-based *agr*P3 reporter via AgrC1 and AgrC1 N267I mutant (a) or AgrC1 Y251F mutant (b), respectively. (c) Bioluminescent *agrP3-lux* reporter expression profiles for strains MY15 and MY20 carrying AgrC1 or AgrC1 N267I, respectively, with or without the addition of AIP-1 or (Ala^5^)AIP-1 at the time of inoculation. All assays were performed in triplicate; error bars represent the SEM. OD, optical density; RLU, relative light units.

To determine whether the cytoplasmic domain AgrC N267I substitution was responsible for the AIP production profile observed and hence the reduced PVL production in TS13 compared to TS14, site-directed mutagenesis of *agrC* was carried out on the *agr* group 1 bioreporter ROJ143 that produces light in response to exogenous AIP-1 but does not produce endogenous AIPs (11). From the dose-response curves obtained, a 3-fold reduction in AgrC sensitivity to AIP-1 was observed with a 50% effective concentration (EC_50_) of 9.6 ± 1.6 nM for the AgrC-N267I bioreporter compared to 3.4 ± 0.6 nM for the control (*P* < 0.01) (Fig. 4a). In comparison, an AgrC-Y251F mutant reporter did not differ in sensitivity (EC_50_ of 2.8 ± 1.2 nM, *P* > 0.05) (Fig. 4b). Since the bioreporter was based on a multicopy plasmid lacking an *agr* autoinduction loop, the N267I mutation was introduced into an intact *agr* locus that was then transferred by phage transduction to an ectopic chromosomal location in an *agr*-null mutant incorporating an *agr*P3-*lux* reporter in the original *agr* locus. The AgrC-N267I mutant reporter (MY20) showed a marked delay in *agr*P3 expression over the growth curve that could be restored to the wild-type (MY15) profile by exogenous AIP-1 and was completely inhibited by (Ala^5^)AIP-1 (Fig. 4c).

### Do other naturally occurring cytoplasmic domain AgrC mutations impact the AIP production profiles?

**FIG 5:**
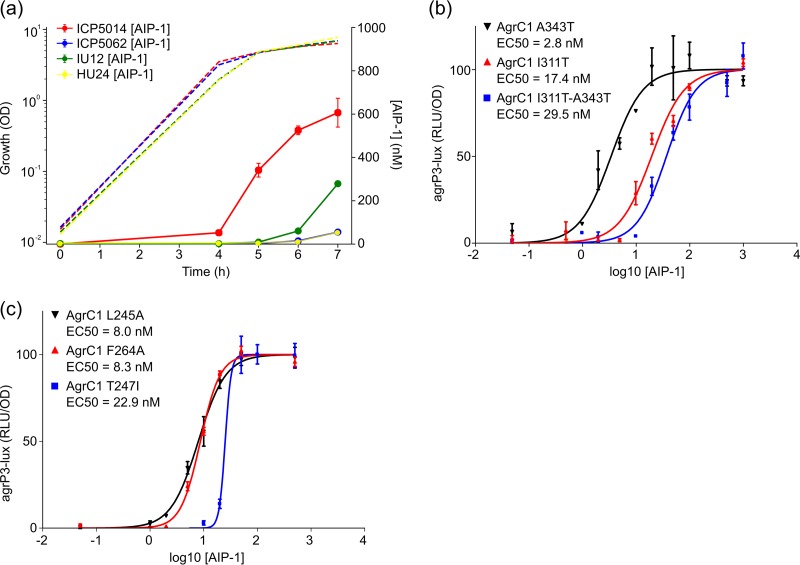
(a) Growth and AIP production for *agr* group 1 strains ICP5014 (WT), ICP5062 (T247I), IU12 (I311T), and HU24 (I311T/A343T). (b) Dose-response curves for the AIP-1-dependent activation of the *lux*-based *agr*P3 reporters incorporating AgrC1 A343T, AgrC1 I311T, or AgrC1 I311T-A343T. (c) Dose-response curves for the AIP-1-dependent activation of *lux*-based *agr*P3 reporters incorporating AgrC1 L245A, F264A, and T247I, respectively. All assays were performed in triplicate; error bars represent the SEM. Hashed lines indicate the growth curves. OD, optical density; RLU, relative light units.

Our previous genome-wide association study of virulence in a collection of S. aureus MRSA ST239 strains identified *agrC* as among the best predictors of cytotoxicity (17). Compared to AgrC from S. aureus strain NCTC8325, all of the isolates from Laabei et al. (17) had an I311T AgrC substitution, with a subset of these also having an A343T substitution. Since strain HU24 (I311T and A343T) was substantially less cytotoxic than strain IU12 (I311T) and the only difference in the *agr* locus was the A343T substitution, we profiled AIP production profiles as a function of growth for both strains. Figure 5a shows that when IU12 is compared to HU24, AIP-1 was produced earlier and at an ∼5-fold higher concentration (279 ± 92 nM compared to 55 ± 79 nM, *P* < 0.01). Introduction of the I311T substitution alone into the ROJ143 *agrP3* reporter increased the EC_50_ from 2.8 ± 1 nM to 17 ± 3 nM (*P* < 0.01), while both I311T and A343T substitutions together increased it significantly further (EC_50_ = 30 ± 3 nM, *P* < 0.01) (17). However, the A343T substitution alone did not significantly affect AgrC sensitivity to AIP-1 (EC_50_ = 3 ± 1 nM; *P* > 0.05) (Fig. 5b).

The *agr* loci of two other ST239 strains lacking the I311T substitution (ICP5014 and ICP5062) but with markedly different cytotoxicities were also compared and found to be identical except for AgrC in ICP5062, which contained a T247I substitution. Although AIP-1 production was undetectable in ICP5062 until the stationary phase (53 nM after 7 h [Fig. 5a]; similar to strain HU24), ICP5014 produced AIP much earlier and reached an ∼10-fold higher concentration (607 nM, *P* < 0.01) (Fig. 5a). The introduction of the T247I substitution into ROJ143, caused a marked reduction in sensitivity to AIP-1 (EC_50_ = 23 ± 3 nM; *P* < 0.01) (Fig. 5c). However, once activated, the AgrC T247I mutant receptor generated a much steeper activation curve than either the wild type or the other *agrC* point mutations evaluated.

Cytoplasmic domain AgrC substitutions in the same region as N267I which have previously been reported to affect exotoxin production include L245S (18) and F264C (12). We substituted both residues, respectively, for Ala in ROJ143 to determine their impact on *agr* function. Figure 5c shows that sensitivity to exogenous AIP-1 was reduced ∼2.5-fold with EC_50_s of 8 ± 2 nM (*P* < 0.05) for L245A and 8 ± 1 nM (*P* < 0.01) for F264A, respectively (Fig. 5c). The comparative data for AIP production and AgrC response in the S. aureus isolates are summarized in Table S5.

### How do the AgrC cytoplasmic domain substitutions impact the structure?

The structure of the AgrC cytoplasmic domain (residues 201 to 430) divided into DHp (dimerization and histidine phosphorylation) and CA (catalytic and ATP binding) subdomains is illustrated in Fig. 1b, which also indicates the locations of the naturally occurring AgrC mutations described above.

**FIG 6:**
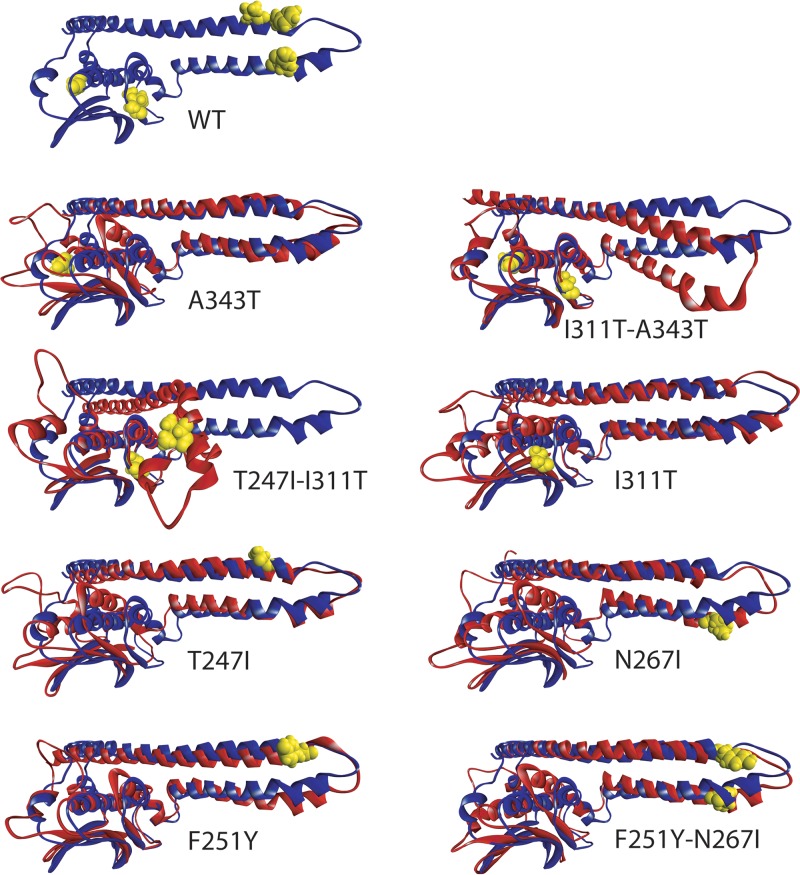
MD-annealed conformation of wild type (WT) AgrC (blue). Side chains are shown in yellow in the WT structure for all mutated residues. Single and double point mutants have been annealed according to an identical protocol and starting structure as for the WT; end structures (red) are shown aligned pairwise to the annealed WT structure (blue). In each pair, mutated residues are shown in full atom representation in yellow for each AgrC substitution shown. Subtle changes near the turn of the helical hairpin translate to markedly reduced accessibility of the ATP-binding pocket (see Fig. 7). Since the WT AgrC already contained a Y251F substitution, this is reversed as F251Y for comparison. In the double mutant T247I-I311T, the helical hairpin buckles and collapses over the CA subdomain, restricting access to the putative AgrA-binding site near the helical turn.

To gain further insights into their impact on domain structure, we generated homology models of the cytoplasmic domains of the AgrC wild type (WT) and F251Y, N267I, I311T, A343T, T247I substituted proteins, as well as the double mutants F251Y-N267I, T247I-I311T, I311T-A343T, and F251Y-N267I (Fig. 6). These include the N-terminal helical hairpin within the DHp subdomain, formed between helix α1 (L205-I252) and helix α2 (M257-N287), which also includes the putative AgrA-binding site near the helical turn, along with a C-terminal CA subdomain (Fig. 1b) in agreement with a previously proposed conformation (19). Since the wild-type AgrC sequence used (NCBI CXR92122) already contained a Y251F substitution, this was reversed for comparison as F251Y for consistency with the strains used. All models were subjected to 80-ns all-atom molecular dynamics simulations using NAMD (20) to determine the impact of the amino acid substitutions on molecular conformation.

Figure 6 shows that the AgrC substitution Y251F in S. aureus strains TS13 and TS14 and the WT AgrC CXR92122 is nearly isomorphic with the F251Y structure, although there is a subtle shift in the helices α1 and α2, as well as in the leading helix α3 of the CA subdomain. However, this shift did not affect the EC_50_ for AgrC activation (Fig. 4b) by AIP-1 in S. aureus ROJ143. The double substitution F251Y-N267I enhances the kink in the trailing helix α2 of the N-terminal hairpin, as observed also in the single mutation N267I, which leads to a very similar conformational change in the C-terminal catalytic domain as for N267I alone (Fig. 6) that resulted in a 3-fold increase in the EC_50_ (Fig. 4a). Both mutations are within the putative AgrA-binding region and, considering the similarity in conformational change in the CA subdomain, the observed change in EC_50_ can be attributed to alteration of the binding site for AgrA resulting from the enhanced kink in helix α2.

AgrC substitutions I311T and A343T reside within the CA subdomain (Fig. 1b). Although I311T is located within the catalytic end of the subdomain, A343T is located directly adjacent to the ATP-binding site (cf. Fig. S2). The conformation of helical hairpin α1-α2 remains almost unperturbed, while a conformational rearrangement is observed in the CA region, including both the catalytic and the ATP-binding subdomains of the A343T mutant (see references 19 and 21). This includes a tilt of helix α3, which is transmitted onto the sheet strands 1 to 5 and is less pronounced in the I311T mutant. However, the ATP-binding domain is affected less in A343T than in I311T, such that in contrast to the A343T substitution, the I311T EC_50_ markedly increases from 3 to 17.4 nM. A striking observation is that the I311T-A343T double mutant shows better alignment of the catalytic domain with the WT paralleled by a significant domain rearrangement between the helical hairpin and the catalytic domain (Fig. 6). The EC_50_ of the double mutant I311T-A343T (29.5 nM) was further increased compared to the single AgrC I311T mutant (17.4 nM). This increase can be attributed to a significant alteration in the AgrA-binding domain where the structural change is most pronounced. Also, in this double mutant, the CA subdomain is brought forward toward the putative AgrA-binding domain and may interfere directly with binding.

The double mutation T247I-I311T is associated with a collapse of the helical hairpin onto the CA subdomain (Fig. 6). This occurs at the end of helix α2 and in the vicinity of I230 within helix α1, where even in the WT we observe an inherent weakness seen as slight uncoiling and bending in helix α1. The location of I230 within helix α1 marks the beginning of the H box (Fig. 1b). Despite this dramatic domain rearrangement and collapse of the helical hairpin into the C-terminal domain, the catalytic domain conformation of this inactive AgrC mutant remains relatively unaffected. However, within this conformation the putative AgrA-binding site is masked by the CA subdomain and would be inaccessible.

Mutations T247I, F251Y, and N267I are located near the turn between helices α1 and α2 directly in the putative AgrA-binding region. Substitutions T247I and N267I kink the long helices α1 and α2, as well as altering their mutual orientation and interactions (Fig. 6). In this respect N267I enhances the kink in the return coil 2, which triggers bending of leading helix α1 at I230 with a coordinated tilt in helix α2 at the loop joining the CA subdomain. This brings the hairpin slightly closer to the CA subdomain, subtly resembling the strong bend observed in the I311T-A343T double mutant. This tilt is accompanied by a subtle shift in helix α3 within the CA subdomain, similar to that observed in A343T (Fig. 6). Mutation T247I has little local effect on the leading helix α1 but affects the supercoiling within the hairpin, which translates into a slight tilt of helix α3 in the CA subdomain, as observed with the N267I mutant.

The observed changes in domain organization within the cytoplasmic domain of AgrC can affect both global and local motional freedom in the polypeptide chain. Changes in local segmental mobility at individual amino acid residues that result from specific mutations, were followed via the root mean square deviation (RMSD) along the trajectory (see Fig. S1 in the supplemental material). Cumulative average excursions over the entire trajectory are summarized in Table S6. Overall, the cytoplasmic domain remains well structured, with little mobility over the majority of the helical hairpin and with cytoplasmic domain and RMSD values remaining within 2.3 to 4.6 Å. Notable flexibility is observed in the loop F386-L395 within the G box and the ATP-binding site, as well as the turn between helix α1 and helix α2 E250-L260.

The local mobility of loop F386-L395 in A343T and I311T-A343T is reduced compared to the WT, whereas I311T alone significantly increases loop mobility. This shows that A343T alone is the deciding factor in restricting local loop dynamics that is sufficient to counter the increase in motional freedom resulting from substitution I311T (Fig. S1). Specifically, flexibility within the WT loop is focused in GLG around L395, whereas in all other cases an increase in loop mobility is observed over a much longer stretch of the chain. Since this loop forms a major part of the ATP-binding pocket, changes in flexibility will likely affect both access of ATP to the binding site and ATPase kinetics.

Mutations N267I just after and T247I just before the helical hairpin turn (Fig. 6) affect both domain conformation and AgrC sensitivity by increasing the EC_50_ by 3- and 7-fold, respectively (Fig. 4 and 5), indirectly by altering the conformation of the helices in the hairpin, as well as directly by altering the binding site for AgrA (19). As the structure anneals throughout the simulation, the loop mobility increases in N267I and to a lesser degree in T247I. One striking observation is that in the double mutant T247I-I311T, the return helix from the hairpin buckles and collapses onto the catalytic domain (Fig. 6). Within the AgrC mutations investigated this was the only case in which a major structural rearrangement of the cytoplasmic domain is observed.

The mutations identified within the cytoplasmic domain of AgrC have a pronounced effect on the mobility of loop E384-G394 located within the G box (Fig. 1b) that correlates with altered activity of the protein. This loop is of critical importance for AgrC function, since on binding to the CA subdomain, an ATP molecule “threads” into the loop to nest against helix α4 and supports the CA subdomain at a fixed position against leading helix α1.

**FIG 7:**
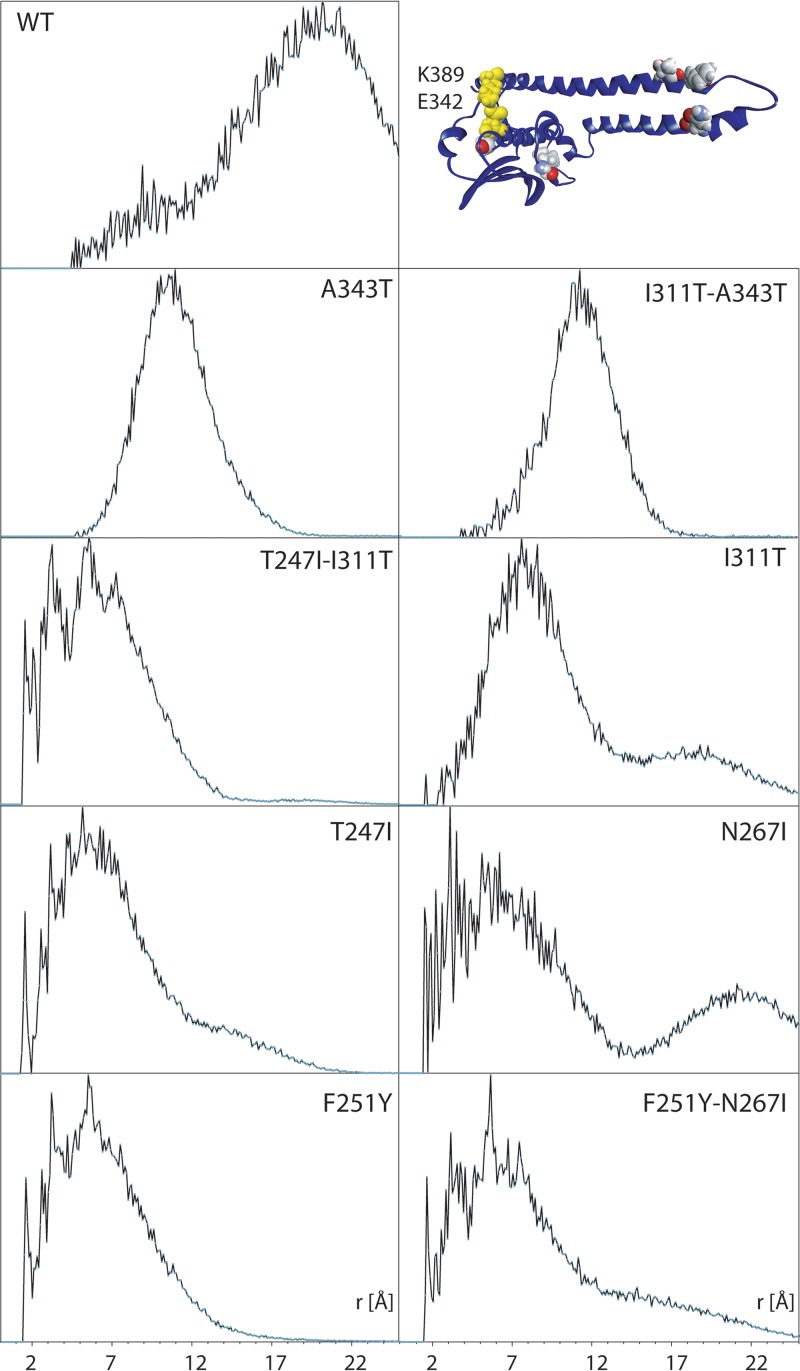
Pairwise RDF *g*(*r*) determined between K389 in the middle of the flexible loop E384-G394 and E342 located helix α4 of the CA subdomain (inset, yellow highlight). The impact of point or double mutations (inset, all atom) is seen as a significant or partial reduction in the average distance/closure of the binding site, which restricts access to the ATP-binding pocket. Mutations N267I and, to a degree, T247I, I311T, and F251Y-N267I show bimodal distribution of access to the ATP-binding site, in which the binding site is open over a small fraction of the trajectory, pointing to reduced binding kinetics.

To assess the impact of these mutations on loop conformation, we used the pairwise radial distribution function (RDF), *g*(*r*), determined between reporter residues K389 in the middle of the loop and E342 located on helix α4 (Fig. 1b and 7). The RDF provides a statistical snapshot of the distance between these residues weighted by the time (number of trajectory frames) the two residues spend at that particular distance (Fig. 7). The RDF in the WT simulation shows a bimodal trajectory occupation with prevalent inter-residue distance near 20 Å corresponding to a more open-loop conformation and a minor population centered near 9 Å corresponding to a closed-loop conformation (Fig. 7). Also, a bimodal trajectory occupation is observed in I311T, but in the reverse occupation with a major closed cluster at 8 Å and a minor open population near 19 Å. Both single A343T and double A343T-I311T mutants show a focused single population centered near 11 and 12 Å, respectively. The fractional population in favor of open or closed states indicates AgrC in a state receptive or refractive to ATP binding, respectively. Both mutations A343T and I311T are located in the CA subdomain and associated with the closed-loop state, in which ATP-binding rates would be reduced.

A bimodal distribution is also observed for mutation N267I in the helical hairpin region with a major closed loop at 6 Å and a parallel, also significant open population at 22 Å. A bimodal loop conformation is also apparent for T247I and F251Y-N267I with a very similar trajectory occupation, a closed conformation at 6Å and a more open conformation at 15Å. Interestingly, both F251Y and T247I-I311T appear closed at 6 Å. All cases of bimodal trajectory occupation observed in Fig. 7 corresponded to higher local flexibility in the E284-G294 loop observed in Fig. 6.

## DISCUSSION

Within a collection of ST22 and ST239 lineage MSSA and MRSA strains we identified a number of point mutations in *agrC* genes that result in delayed AIP production and accumulation and reduced exotoxin but which nevertheless respond to exogenous AIP in a growth-phase-dependent manner. The *agr* system is subject to complex control by a number of regulators, repressors, and alternative sigma factors, including the Sar proteins, Rot, and MgrA (22, 23). We propose that the observed “window period” of responsiveness to exogenous AIP may be influenced by the activity of these alternative regulators, but the mechanism involved is unclear and requires further investigation.

Molecular dynamics simulations from *in silico*-engineered point mutations in AgrC cytoplasmic domain revealed subtle changes in the immediate vicinity of the amino acid substitutions that altered the individual domain conformation, as well as the relative domain orientation. Nonadjacent double mutations had markedly amplified consequences for the structural stability of the domain. An indirect consequence of the cytoplasmic domain mutations was the altered accessibility, mobility, and conformation of ATP-binding loop E384-G394 in the G box of the CA subdomain.

The key AgrC substitution in TS13, i.e., N267I, distorted packing of the leading α-helices in the dimerization domain independently of the presence of Y251F, which alone also changed orientation of the catalytic domain with respect to the helical hairpin dimerization interface (Fig. 6). This mutation may either impair dimerization or impede the autophosphorylation of H239 during activation. Surprisingly, the other key mutation in the dimerization region, T247I, did not appear to alter dramatically the structure of the cytoplasmic domain despite an observed ∼10-fold increase in EC_50_ in the reporter strain, which suggests that this mutation is more likely to disrupt dimer assembly in the functional AgrC domain or directly interfere with binding of AgrA. In contrast, in the double mutant T247-I311T, the dimerization helical hairpin folded and collapsed onto the catalytic domain (Fig. 6). Given the proximity of T247I to H239, this mutation is likely to interfere with dimerization, as well as disrupt access to the catalytic subdomain during autophosphorylation, thereby impacting the efficiency of phosphotransfer to AgrA. Hydrophobic residues at AgrC positions 242, 244, 245, and 248 were previously noted to be involved in AgrC-AgrA interactions by (19), although substituting the Y251 for W did not substantially alter phosphotransfer kinetics.

AgrC has been suggested to behave like a rheostat, where activation of its membrane-spanning domain on binding a cognate AIP results in the twisting of a helical linker relative to the cytoplasmic domain and subsequent dimerization (9). We propose that the substitutions observed here in the dimerization region reduce the efficiency of this process, requiring a greater magnitude of “input” into the rheostat-like mechanism in order to produce the same response. This would in turn impact the efficiency of the autoinduction circuitry. Consequently, we examined the impact of the AgrC mutations reported here on the noncovalent interaction between R238 and Q305 shown by Xie et al. (10) to be associated with maintaining AgrC in its inactive state. While in the WT domain structure these residues are separated by approximately 20 Å, the collapse of helix α1 at I230, which is most pronounced in the T247I-I311T and I311T-A343T double mutants, brings the R238 and Q305 residue pair closer together, which, combined with a stochastic process, can result in a H-bond-stabilized closed conformation. This may explain the requirement for higher concentrations of AIP that would facilitate the longer receptor occupancy needed to generate sufficient force to twist the helical linker and break the latch to activate *agr*.

We also examined the impact of point mutation Y223C in helix α1, which was reported to decrease downstream signaling (15). MD simulations showed that Y223 is responsible for stabilizing the relative orientation between helix α1 and the CA subdomain. The Y223C mutation leads to a slip of the catalytic domain past helix α1, supercoiling of the hairpin with the concomitant, but partial closure of the reporter pair E324/K389 from 17 Å in the WT to 12 Å in Y223C.

Data from cytotoxicity, AIP, and mutant bioreporter assays collectively suggest that A343T and I311T have a cumulative effect on *agr* function. Although I311T has the greater impact on the orientation of the mobile loop in the CA subdomain and overall RMSD variation (4.8 ± 1.2 Å) (Table S6 and Fig. S1), the double mutant I311T-A343T resulted in a major repositioning of the dimerization hairpin with respect to the CA subdomain. Altered accessibility to the ATP-binding site, modulated by the flexibility and/or bistable conformational preference of loop E384-G394, could impact ATP binding as well as the efficiency of autophosphorylation, thus explaining the altered *agr* activity observed in the clinical isolates harboring the double mutation.

The conformational impact of point mutations in the cytoplasmic domain of AgrC appear delocalized, cooperative, and distributed over the entire domain, potentially affecting catalytic activity, dimerization, and downstream AgrA-mediated signaling. This points to a role for the cytoplasmic domain of AgrC as a virulence modulator rather than as a binary switch. Such fine-tuning in signal-mediated gene regulation provides evolutionary guidance and an opportunity for adaptation in response to environmental changes rather than serving as a definitive and committed survival response.

This conformational analysis of point mutations in cytoplasmic AgrC, combined with studies of the whole protein (19), a chimera with the leucine zipper GCN4 (10), and other TCS (21), suggests a model in which a metastable supercoil between helices α1 and α2 is stabilized by ATP-dependent interactions with the CA domain and modulated by a twist of helix α1 driven by the sensory membrane domain. Fine-tuning involves allosteric structural modulation of the accessibility to the ATP-binding site and ATPase kinetics, as well as accessibility and binding of the downstream response element AgrA.

The naturally occurring, single-amino-acid-change, cytoplasmic AgrC substitutions studied here reduced *agr* activity without completely abolishing activation. As a consequence, they could be seen as an ideal adaptation of clinical strains to host niches where high-level exotoxin production is not required but where some measure of flexibility would be useful. Interestingly, each of the AgrC substitutions investigated in the present study is dependent on a single base change such that reversion to the wild type would be readily selectable. It has also been hypothesized that *agr* mutants show reduced fitness for transmissibility or possibly the establishment of new infections (13–15). Alternatively, the reduced *agr* phenotype could promote further cell and tissue adherence or facilitate intracellular access during established infections.

This study demonstrates how naturally occurring AgrC mutations impact *agr* activation kinetics and exotoxin production with reference to the structure and function of the AgrC. Future studies will need to address how the temporospatial and environmental factors present during *in vivo* infection variably effect isolates with or without attenuated *agr* functionality. Understanding the potential benefit of such variations could be key to understanding the development of acute infection in established colonization or chronic infections, such as recurrent abscesses or foot ulcers, and further delineate the potential role for antivirulence therapies targeted at QS in S. aureus.

## MATERIALS AND METHODS

### Bacterial strains, plasmids, and culture conditions.

The bacterial strains and plasmids used in this study are described in Table S1 in the supplemental material. S. aureus strains were grown aerobically in brain heart infusion (BHI) or CYGP broth (24) at 37°C with shaking at 250 rpm. Where required, growth media were supplemented with erythromycin (10 μg/ml), chloramphenicol (10 μg/ml), or tetracycline (10 μg/ml). The oligonucleotides used in the study are listed in Table S2 in the supplemental material. AIP-1, AIP-3, and (Ala^5^)AIP-1 were synthesized as previously described (24). For *agr* activation and inhibition experiments, a 100 nM concentration of the required AIP was added to the culture medium prior to inoculation.

### *S. aureus* genome sequencing.

Genomic DNA was prepared from S. aureus TS13 and TS14 using a DNeasy kit (Qiagen) and sequenced with an Illumina MiSeq 2 × 150-bp paired-end run. Reads were mapped to the EMRSA-15 representative genome HO 5096 0412 (GenBank accession no. NC_017763.1) (25) with the Burrows-Wheeler alignment tool (26). SNPs were identified with mpileup and bcftools (27) using default settings and filtering out base quality scores lower than 15 and SNPs present in fewer than five reads. A list of the genes containing variants unique to either TS13 or TS14 was compiled using Artemis 14.0.0 software (Wellcome Trust Sanger Institute, Cambridge, United Kingdom). The whole-genome sequencing data for TS13 (ENA accession no. ERS3409631) and TS14 (ENA accession no. ERS3409632) were deposited in the European Nucleotide Archive under ENA study accession no. PRJEB32550 (https://www.ebi.ac.uk/ena).

### AIP bioreporter construction and site-specific mutagenesis of *agrC*.

S. aureus ROJ143 is an *agr*P3::*lux* bioreporter strain constructed by replacing the entire *agr* locus in RN4220 with the erythromycin resistance gene *ermB* and an *agrP3*::*luxABCDE* promoter and transformed with plasmid pAgrP2C1A, containing the *agr*P2 promoter, *agrC1*, and *agrA* (11). This bioreporter is incapable of producing AIP but produces light in response to exogenous AIP-1. Modified bioreporters, with specific *agrC* mutations, were constructed by site-specific mutagenesis of the pAgrP2C1A plasmid. Mutagenesis was performed using the phosphorylated primers shown in Table S2 and Phusion DNA polymerase (New England Biolabs, United Kingdom) before ligation of the resulting PCR products by Quick Ligase enzyme (New England Biolabs). The plasmids carrying the mutated *agrC1* genes were introduced into ROJ48 (11).

### Chromosomal *agr* mutant construction.

The S. aureus strains MY15 and MY20 (see Table S1 in the supplemental material), harboring the *agr* locus at an ectopic chromosomal site, were constructed by phage transduction. Plasmid pEJM6 was made by introducing the wild-type group 1 *agr*P2*BDCA* genes amplified with primers EJM63 and EJM65 (Table S2) into the integrative plasmid pLL102 (Table S1). pEJM6 was transformed into a chromosomal integration strain of S. aureus CYL12349 (28), resulting in the integration of *agrP2BDCA* at an *attB*2 site on the CYL12349 chromosome. Phage lysate prepared from the latter was mixed with helper Φ11 and used to transduce the Δ*agr*::*agrP3-lux* bioreporter ROJ48 to generate MY15. MY20 was constructed by first introducing *agrC* N267I constructed by site directed mutagenesis into pEJM6, to make pMYP3, before integrating into CYL12349 and transducing into ROJ48 as above.

### PVL immunoblotting.

Exoproteins from S. aureus culture supernatants grown in CYGP broth were precipitated with trichloroacetic acid (10% [vol/vol]) after normalizing to the same optical density at 600 nm. Precipitated exoproteins were separated on 12% (wt/vol) SDS-PAGE gels, transferred to a nitrocellulose membrane, and blocked in 5% (wt/vol) skimmed milk in phosphate-buffered saline (pH 7.4) prior to incubation with mouse monoclonal antibodies to PVL, followed by a protein A-horseradish peroxidase conjugate (Sigma) and developed using ECL substrate (GE Healthcare).

### Quantification of PVL and RNAIII transcription by qRT-PCR.

S. aureus TS13 and TS14 were grown in BHI broth until mid-exponential phase. RNA Protect (Qiagen) was added, the cells were lysed and RNA purified using an RNeasy minikit (Qiagen). cDNA was synthesized from RNA samples with Superscript II reverse transcriptase (Invitrogen) and amplified using a Power SYBR green master mix (Life Technologies Corp.) using the required forward and reverse primers (Table S2). Amplification was recorded using an AB7500 real-time PCR system (Applied Biosystems), and comparative cycle threshold values were calculated to determine the relative expression of the target genes *lukS-PV* and RNAIII, with *gyrB* as a constitutively expressed control.

### Bioluminescence assays and AIP quantification.

Lux-based bioreporters containing either wild-type or mutant agrP2C1A plasmids (Table S1) were grown overnight at 37°C in BHI with 10 μg/ml chloramphenicol, diluted 1:50 in fresh BHI, and grown for a further 2 h prior to dilution to 1:20 into a 96-well microtiter plate containing triplicate serial dilutions of exogenous AIPs. Bioluminescence was quantified in a Tecan microplate reader, and data were plotted as relative light units/optical density (RLU/OD) over time. Peak values from each concentration of AIP were exported to Prism 2 (GraphPad, San Diego, CA), and EC_50_ values were generated from sigmoidal dose-response curves.

To quantify relative AIP concentrations in S. aureus cultures throughout growth in BHI, samples were removed at specific time points and incubated with the bioluminescent *agr* reporter strain ROJ143 (Table S1), and the relative AIP concentration was determined from a calibration curve generated using a range of synthetic AIP standards.

### Homology modeling and molecular mechanics simulations.

The sequence of AgrC from group 1 S. aureus is identical to UniProt accession no. Q2VG18 and NCBI GenPept accession no. CXR92122. Structural information is unavailable for full-length AgrC membrane and cytoplasmic domains modeled separately, and only the C-terminal catalytic part of the cytoplasmic domain has been characterized (19). We used I-TASSER (29) and Phyre2 (30) to obtain a model of the cytoplasmic domain of AgrC residues L204-N430. Specifically, modeling templates included the AgrC catalytic C-terminal domain from S. aureus (PDB 4BXI), tyrosine kinase from Caulobacter crescentus (PDB 4Q20), and C. vibrioides (PDB 5IDJ).

The models were in good agreement and were subsequently annealed by all atom molecular dynamics for 60 ns using NAMD (20) with the CHARMM 36 forcefield. In brief, the I-TASSER model was prepared in CHARMM (31) using CHARMM-GUI (32). It was hydrated in a rectangular water box that included 150 mM KCl while maintaining 35-Å separation from its images, generated by the periodic boundary conditions to preclude overlap of the protein/image Debye layers at this ionic strength. The model was annealed by heating from 0 to 303 K, and 10-ns short production runs were carried out on a Supermicro U1 server equipped with NVIDIA Tesla GPU accelerators for parallel vector calculations. The annealed end states were transferred for parallel scalar calculations to the MidlPlus or to the University of Nottingham High Performance Computing facility, where the longer production runs were obtained from sequential 40-ns trajectories. The trajectories were concatenated using catdcd, a module of VMD (33). Trajectory analysis was done using VMD (33), and molecular visualization and model alignment were done using Discovery Studio (Biovia; Dassault Systèmes). The end state was used as a starting point for the *in silico* mutagenesis and subsequent comparative analysis. Wild type was prepared for MD simulations and, in parallel, the annealed model was mutated in CHARMM-GUI.

The docked conformation of ATP in the cytoplasmic domain of AgrC was obtained from exploratory semirigid docking using Swiss-Dock (34), and the top ranking binding site was found in agreement with flexible docking using Autodock Vina (35). Molecular visualization was done in UCSF Chimera (36).

### Data availability.

The Staphylococcus aureus reference genomes used include HO 5096 0412 (GenBank accession no. NC_017763.1) and NCTC8325 (GenBank no. CP000253.1). Whole-genome sequencing data for this study are available in the European Nucleotide Archive (ENA no. PRJEB32550) for TS13 (ENA no. ERS3409631) and TS14 (ENA no. ERS3409632). AgrC protein sequences were accessed from UniProt (UniProt no. Q2VG18) and the NCBI Peptide Database (GenPept no. CXR92122). Crystal structures were obtained from the Protein Data Bank, including AgrC from S. aureus (PDB 4BXI), tyrosine kinase from Caulobacter crescentus (PDB 4Q20) and *C. vibrioides* (PDB 5IDJ).

## Supplementary Material

Supplemental file 1
