# Supplementary material for: Timing Is Everything: Impact of Naturally Occurring Staphylococcus aureus AgrC Cytoplasmic Domain Adaptive Mutations on Autoinduction
Source: J Bacteriol. 2019 Sep 20;201(20):e00409-19. doi: 10.1128/JB.00409-19 (PMC6755735; doi:10.1128/JB.00409-19)
Supplement: Supplemental file 1 [file JB.00409-19-s0001.pdf]

Supplementary Information

Table S1. Bacterial strains and plasmids used in the study.

Name	Description	Source
S. aureus		
RN4220	8325-4 restriction negative derivative *rsbU-*	Kreiswerth et al 1983 (1)
ROJ48	RN4220 Δagr::*ErmB agr*P3-*lux* Tet^R Erm^R	Jensen et al 2008 (2)
ROJ143	ROJ48 pAgrP2C1A	Jensen et al 2008 (2)
TS112	ROJ48 pAgrP2C1A *agrC N267I*	This laboratory
TS113	ROJ48 pAgrP2C1A *agrC I311T*	Jensen et al 2008 (2)
TS114	ROJ48 pAgrP2C1A *agrC Y251F*	This laboratory
TS115	ROJ48 pAgrP2C1A *agrC A343T*	This laboratory
TS119	ROJ48 pAgrP2C1A *agrC N267I Y251F*	This laboratory
TS120	ROJ48 pAgrP2C1A *agrC I311T A343T*	This laboratory
TS143	ROJ48 pAgrP2C1A *agrC L245A*	This laboratory
TS144	ROJ48 pAgrP2C1A *agrC F264A*	This laboratory
TS145	ROJ48 pAgrP2C1A *agrC N308A*	This laboratory
TS152	ROJ48 pAgrP2C1A *agrC T247I*	This laboratory
CYL12349	RN4220 *attB2* pYL112Δ19	Lei et al 2012 (3)
MY15	ROJ48 *attB2*::*P2agrC1agrA*	This laboratory
MY20	ROJ48 *attB2*::*P2agrC1agrA* AgrC-N267I	This laboratory
TS12	ST30 MSSA spa t021 PVL+, wound infection	Okokunefor et al 2012 (4)
TS13	ST22 MSSA spa t005 PVL+, leg abscess	Okokunefor et al 2012 (4)
TS14	ST22 MSSA spa t005 PVL+, leg ulcer	Okokunefor et al 2012 (4)
IU12	ST239 *agrC I311T*	Laabei et al 2014 (5)
HU24	ST239 *agrC I311T A343T*	Laabei et al 2014 (5)
ICP5014	ST239	Laabei et al 2014 (5)
ICP5062	ST239 *agrC T247I*	Laabei et al 2014 (5)
**Plasmids**		
pAgrP2C1A	pSKerm *agr*P2 *agrC1 agrA* Cm^R	Jensen et al 2008 (2)
pLL102	Derivative of pCL25 carrying *attP2* Tet^R	Lei et al 2012 (6)
pEJM6	pLL02 *P2agrC1agrA* Tet^R, Kan^R	This laboratory
pMYP3	pLL102 *P2agrC1agrA* AgrC-N267I Tet^R, Kan^R	This laboratory

MSSA = Methicillin sensitive *S. aureus*. MRSA = Methicillin resistant *S. aureus*. NUH = Nottingham University Hospitals. CC = Clonal complex. ST = Sequence type. PVL = Panton-Valentine Leukocidin. Tet^R = Tetracycline resistance. Erm^R = Erythromycin resistance. Cm^R = Chloramphenicol resistance. Kan^R = Kanamycin resistance.

Table S2. Oligonucleotides used in the study

Name	Sequence (5' to 3')	Application
AgrBDCAF	ACAACTCATCAACTATTTTCC	*agr* amplification
AgrBDCAR	GATTTACAATTGAATACGCCG	*agr* amplification
Agrfull F	GCAACGCGAAAATATACCTG	*agr* sequencing
Agrfull R	GCGTTGCTGCAATAGTGACA	*agr* sequencing
AgrC 5' B	AAAGAGATGAAATACAAACG	*agr* sequencing
AgrA 5' A	ATTAACAACTAGCCATAAGG	*agr* sequencing
AgrB F	AATAAAATTGACCAGTTTGC	*agr* sequencing
agrC R	GAATAATACCAATACTGCGAC	*agr* sequencing
AgrC-N267I-F	(P)AGACAATTTACAAATGAATGCTATAAAATTA	SDM
AgrC-N267I-R	(P)TTCATAGGTACAATAATTTTATTGAAATAAT	SDM
AgrC-Y251F-F	(P)CACTTTCAGAATTCATTCGAGAAGATGA	SDM
AgrC-Y251F-R	(P)TCGTTAAGATATTGACATAATCATGACG	SDM
AgrC-A343T-F	(P)GATAATGCAATTGAGACATCAACTGAAA	SDM
AgrC-A343T-R	(P)AAGAATAATACCAATACTGCGACTTAAATC	SDM
AgrC-I311T-F	(P)AAATGAATATTCCGACTAGTATCGAAATACC	SDM
AgrC-I311T-R	(P)CTTGTGCACGTAAAATTTTCGCAGTAAT	SDM
AgrC-L245A-F	(P)ATTATGTCAATATCGCAACGACACTTTCAG	SDM
AgrC- L245A-R	(P)CATGACGGAACTTGCGCA	SDM
AgrC-F264A-F	(P)GGCCTACGTGATTATGCTAATAAAAATATTGTACC	SDM
AgrC- F264A-R	(P)AGGCATGTCATCTTCTCGAATGTA	SDM
AgrC-N308A-F	(P)GCACAAGAAATGGCTATTCCGATTAGTATC	SDM
AgrC- N308A-R	(P)ACGTAAAATTTTCGCAGTAATTAAGCCTT	SDM
AgrC- T247I-F4	(P)TACTTTCAGAATACATTCGAGAA	SDM
AgrC- T247I-R4	(P)TCGTTAAGATATTGACATAATCAT	SDM
GyrB F	GGTGGCGACTTTGATCTAGC	qRT-PCR
GyrB R	TTATACAACGGTGGCTGTGC	qRT-PCR
LukS-PV F	AATAACGTATGGCAGAAATATGGATGT	qRT-PCR
LukS-PV R	CAAATGCGTTGTGTATTCTAGATCCT	qRT-PCR
RNAIII F	GGGATGGCTTAATAACTCATAC	qRT-PCR
RNAIII R	GGAAGGAGTGATTTCAATGG	qRT-PCR
EJM63	ATACGAATTCCCATGGCAACTATTTTCCATCACA	*agr* cloning
EJM65	ATCGGATCCGTTAACTGACTTTATTATC	*agr* cloning

(P) = 5' phosphorylation. (B) = 5' biotinylation. SDM = site directed mutagenesis. qRT-PCR = quantitative real time PCR.

Table S3. Non-synonymous SNPs found in TS13 only

A complete list of SNPs unique to TS13 that resulted in coding sequence changes. The location numbers indicate the base position on the reference strain HO 5096 0412 (GenBank accession no. **NC_017763.1**) with both the reference base and TS13 variant shown. The coding sequence ID from the reference annotation is in column 4 with the annotated gene name (if known) in column 5 and description in column 6. The SNPs in *agrC* (N267I) and *sarU* (D134Y) are shown in bold.

Location	Reference	TS13	Coding Sequence ID	Gene product	Description
116754	G	A	920	-	putative_membrane_protein
116755	G	A	920	-	putative_membrane_protein
182155	G	A	1480	argJ	putative_arginine_biosynthesis_bifunctional_protein
296240	G	A	2370	-	putative_membrane_protein
303593	G	A	2420	-	putative_membrane_protein
352330	C	G	2880	-	putative_PTS_multidomain_regulator
413095	G	A	3490	-	hypothetical_protein
440311	G	T	3770	-	putative_NADH-Ubiquinone/plastoquinone_(complex_I)_protein
458517	C	T	3960	-	LysR-family_regulatory_protein
499087	G	A	4280	-	putative_peptidyl-tRNA_hydrolase
546662	G	A	4570	-	conserved_hypothetical_protein
594078	T	C	4930	-	conserved_hypothetical_protein
637356	G	A	5400	-	putative_hydrolase
676430	C	G	5800	-	putative_membrane_protein
714778	C	T	6180	-	putative_membrane_protein
736167	T	C	6410	-	putative_para-aminobenzoate_synthetase_component
933720	A	G	8230	-	putative_oligopeptide_transport_system_permease_protein_(pseudogene)
1044535	C	T	9260	pdhD	dihydrolipoamide_dehydrogenase
1074548	G	A	9580	isdB	iron-regulated_heme-iron_binding_protein_(pseudogene)
1243331	T	C	11110	-	conserved_hypothetical_protein
1253375	A	G	11200	-	transposase_(fragment)

1256697	A	G	11240	-	putative_pyruvate_flavodoxin/ferredoxin_oxidoreductase
1314907	A	G	11870	-	putative_membrane_protein
1322367	T	A	11930	-	putative_exonuclease
1384978	G	C	12500	-	ABC_transporter_ATP-binding_protein
1449776	C	T	12970	ebh	very_large_surface_anchored_protein_(pseudogene)
1481909	C	A	13120	-	putative_exported_protein
1605227	T	A	14460	-	conserved_hypothetical_protein
1606147	G	A	14470	accC	biotin_carboxylase_subunit_of_acetyl-CoA_carboxylase
1638949	A	G	14840	-	conserved_hypothetical_protein,_pseudogene
1663284	A	T	15070	-	putative_membrane_protein
1713713	T	A	15560	tgt	queuine_tRNA-ribosyltransferase
1827886	T	C	16530	-	putative_aminopeptidase
1853199	C	T	16710	-	transposase
1881489	T	G	16990	pckA	phosphoenolpyruvate_carboxykinase
1883817	C	A	17020	-	conserved_hypothetical_protein
1924033	G	A	17480	-	putative_membrane_protein
1995463	C	A	18090	putP	high_affinity_proline_permease
2007441	A	G	18180	-	transposase_(fragment)
2007881	C	A	18190	-	transposase_(fragment)
2007902	T	C	18190	-	transposase_(fragment)
2017193	C	T	18280	-	isochorismatase_family_protein
2027595	T	A	18370	-	Tn554-related,_transposase_B
2035006	C	T	18470	-	putative_ABC_transporter_ATP-binding_protein
2038477	T	C	18510	-	hypothetical_protein
**2108636**	**A**	**T**	**19460**	**agrC**	**autoinducer_sensor_protein**
2160133	G	A	19870	kdpA	putative_potassium-transporting_ATPase_a_chain
2293253	G	A	21030	-	hypothetical_protein
2405583	G	A	22290	hutU	urocanate_hydratase
2424120	G	A	22480	-	hypothetical_protein
2492551	A	T	23120	-	transport_system_membrane_protein

2509829	G	A	23280	-	transposase_(fragment)
2509877	G	A	23280	-	transposase_(fragment)
2510039	A	G	23280	-	transposase_(fragment)
2540395	T	G	23510	-	putative_carboxylesterase
2584539	G	T	23940	-	putative_membrane_protein
2586266	C	T	23950	sasG	LPXTG_surface_protein_(pseudogene)
2586704	A	G	23950	sasG	LPXTG_surface_protein_(pseudogene)
**2589488**	**G**	**T**	**23970**	**sarU**	**staphylococcal_accessory_regulator**
2595084	G	A	24010	gntP	putative_gluconate_permease
2597763	T	C	24030	gntR	gluconate_operon_transcriptional_repressor
2608565	G	T	24120	-	ABC_transporter_ATP-binding_protein
2614629	G	T	24170	-	putative_dioxygenase
2639555	G	A	24450	-	LysR-family_regulatory_protein
2645865	A	G	24510	clpL	putative_ATP-dependent_protease_ATP-binding_subunit_ClpL
2664626	G	T	24660	-	putative_glycosyltransferase
2666092	C	T	24670	-	putative_phytoene_dehydrogenase_related_protein
2737433	G	A	25350	clfB	fibrinogen_and_keratin-10_binding_surfaced_anchored_protein
2737646	T	C	25350	clfB	fibrinogen_and_keratin-10_binding_surfaced_anchored_protein
2737658	A	G	25350	clfB	fibrinogen_and_keratin-10_binding_surfaced_anchored_protein
2737661	A	G	25350	clfB	fibrinogen_and_keratin-10_binding_surfaced_anchored_protein
2737673	G	A	25350	clfB	fibrinogen_and_keratin-10_binding_surfaced_anchored_protein
2737685	A	G	25350	clfB	fibrinogen_and_keratin-10_binding_surfaced_anchored_protein
2791513	A	G	25750	icaB	intercellular_adhesion_protein_B
2810175	T	C	25930	cna	collagen_adhesin_precursor
2810187	A	G	25930	cna	collagen_adhesin_precursor
2810190	T	C	25930	cna	collagen_adhesin_precursor
2810331	C	T	25930	cna	collagen_adhesin_precursor

Table S4. Non-synonymous SNPs found in TS14 only

A complete list of SNPs which were unique to TS14 and resulted in coding sequence changes. The location numbers indicate the base position on the reference strain HO 5096 0412 (GenBank accession no. **NC_017763.1**) with both the reference base and TS14 variant shown. The coding sequence ID from the reference annotation is in column 4 with the annotated gene name (if known) in column 5 and description in column 6.

Location	Reference	TS14	Coding Sequence ID	Gene product	Description
36913	G	T	270	-	putative_transposase
68139	A	T	520	-	TetR_family_regulatory_protein
68141	A	C	520	-	TetR_family_regulatory_protein
131435	T	C	1070	-	binding-protein-dependent_transport_system_membrane_component
166233	G	A	1380	-	putative_lipoprotein
168047	T	G	1400	-	conserved_hypothetical_protein,_pseudogene
271844	C	T	2160	-	hypothetical_protein
284663	G	A	2260	bglA	6-phospho-beta-glucosidase
361479	C	A	2960	-	putative_membrane_protein
382759	A	G	3160	-	putative_membrane_protein
540747	C	T	4520	radA	putative_DNA_repair_protein
561505	A	G	4720	rpsL	30S_ribosomal_protein_S12
688820	G	A	5910	-	putative_exported_protein
691792	C	T	5930	-	AraC_family_regulatory_protein
704056	A	C	6090	bacA	putative_undecaprenol_kinase
720120	C	T	6250	-	putative_phosphofructokinase
742346	G	A	6460	-	ABC_transporter_ATP-binding_protein
787395	C	T	6850	uvrA	excinuclease_ABC_subunit_A
878668	A	G	7770	mnhF	Na+/H+_antiporter_subunit
886859	G	A	7860	-	NADH:flavin_oxidoreductase_/_NADH_oxidase_family_protein
963079	G	A	8500	prfC	peptide_chain_release_factor_3
1015251	C	T	9000	purL	putative_phosphoribosylformylglycinamidine_synthase_II

1125117	C	T	10100	-	conserved_hypothetical_protein
1143560	A	G	10270	-	glyoxalase/bleomycin_resistance_protein/dioxygenase_superfamily_protein
1158601	G	A	10410	-	conserved_hypothetical_protein
1224943	G	T	10970	polC	DNA_polymerase_III_PolC-type
1228731	C	T	10990	nusA	putative_N_utilization_substance_protein_A
1253708	C	A	11200	-	transposase_(fragment)
1271854	G	A	11360	-	putative_hydrolase
1351231	A	T	12150	trpE	anthranilate_synthase_component_I
1432637	C	T	12970	ebh	very_large_surface_anchored_protein_(pseudogene)
1567552	G	A	13480	-	hypothetical_protein,_pseudogene
1589216	C	G	14310	-	glyoxalase/bleomycin_resistance_protein/dioxygenase_superfamily_protein
1652227	A	G	14990	dnaJ	chaperone_protein
1673889	T	C	15210	-	hypothetical_phage_protein
1686714	G	C	15360	alaS	putative_alanyl-tRNA_synthetase
1705223	C	T	15510	relA	GTP_pyrophosphokinase
1851017	C	A	16680	-	conserved_hypothetical_protein
1883618	C	T	17020	-	conserved_hypothetical_protein
1916224	G	A	17390	trap	signal_transduction_protein
2162741	G	T	19880	kdpD	sensor_kinase_protein
2177589	T	C	20030	tenA	transcriptional_activator
2178861	C	T	20040	-	putative_exported_protein
2235170	A	G	20620	mtlA	PTS_system,_mannitol-specific_IIBC_component
2240552	C	G	20660	fmtB	putative_surface_anchored_protein
2249903	T	C	20690	-	putative_membrane_protein
2477430	G	A	22960	narG	nitrate_reductase_alpha_chain
2529128	T	C	23420	-	hypothetical_protein
2586650	C	T	23950	sasG	LPXTG_surface_protein_(pseudogene)
2592008	C	A	23990	fnbA	fibronectin-binding_protein_FnbA
2632747	A	G	24370	-	transposase_(pseudogene)
2634271	A	G	24410	glcB	PTS_system,_glucose-specific_IIABC_component

2650324	T	C	24550	-	putative_transport_protein
2657496	G	A	24600	copA	putative_copper_importing_ATPase_A
2673509	T	C	24730	-	putative_membrane_protein
2682118	G	A	24840	-	alcohol_dehydrogenase
2702279	T	G	25060	ldh2	L-lactate_dehydrogenase_2
2737436	T	G	25350	clfB	fibrinogen_and_keratin-10_binding_surfaced_anchored_protein
2810244	G	A	25930	cna	collagen_adhesin_precursor
2810302	G	C	25930	cna	collagen_adhesin_precursor
2810622	G	A	25930	cna	collagen_adhesin_precursor
2810673	A	G	25930	cna	collagen_adhesin_precursor
2810748	G	A	25930	cna	collagen_adhesin_precursor
2810781	A	G	25930	cna	collagen_adhesin_precursor

Table S5. Summary of AIP production and AgrC activation by clinical *S. aureus* strains

Summary of AIP production profiles of paired clinical strains analysed throughout growth. Strains are shown with corresponding AgrC substitutions in brackets. The time when AIP was first detected is indicated with the mean final AIP concentrations shown in nM (SD). EC_{50} values for the corresponding *agrC* mutations when introduced into an *agrC1A*-*agrP3*::*lux* bioreporter strain are also shown in nM (SD). Growth rate (μ) was calculated as ln2/doubling time (h) during exponential phase.

Strain (AgrC substitution)	Timing of AIP induction (h)	Stationary AIP concentration (nM +/- SD)	Bioreporter EC_{50} (nM +/- SD)
TS14 (Y251F)	3.5	1052.5 (40.2)	2.8 (1.2)
TS13 (Y251F/N267I)	4.5	546.4 (32.3)	9.3 (1.8)
IU12 (I311T)	6.0	278.8 (92.3)	17.4 (3.5)
HU24 (I311T/A343T)	7.0	55.1 (7.8)	29.5 (3.1)
ICP5014 (WT)	4.0	606.6 (115.1)	3.4 (0.6)
ICP5062 (T247I)	7.0	53.4 (9.7)	22.9 (2.6)

Table S6. RMSD values comparing the WT to AgrC mutants

	RMSD Å (SD)
WT	3.0 (0.5)
A343T	2.6 (0.8)
I311T	4.8 (1.2)
I311T-A343T	3.5 (0.6)
N267I	3.2 (0.9)
T247I	2.6 (0.7)
T247I-I311T	8.3 (1.5)
F251Y	2.6 (0.8)
F251Y-N267I	3.3 (1.0)

Figure S1. Heatmap plots of AgrC mutant and wild type proteins showing the root mean square deviation (RMSD) variation for each residue along the chain (ordinate) over an 80 ns trajectory (abscissa blocks). RMSD values are colour-encoded according to the colour chart on the right up to 25Å. In mutants T247I-I311T and F251Y-N267I maximum variation is 41.1 and 26.5Å, respectively, and extremes are colour saturated. All mutants are plotted on the same colour scale. RMSD analysis was done using VMD (7).

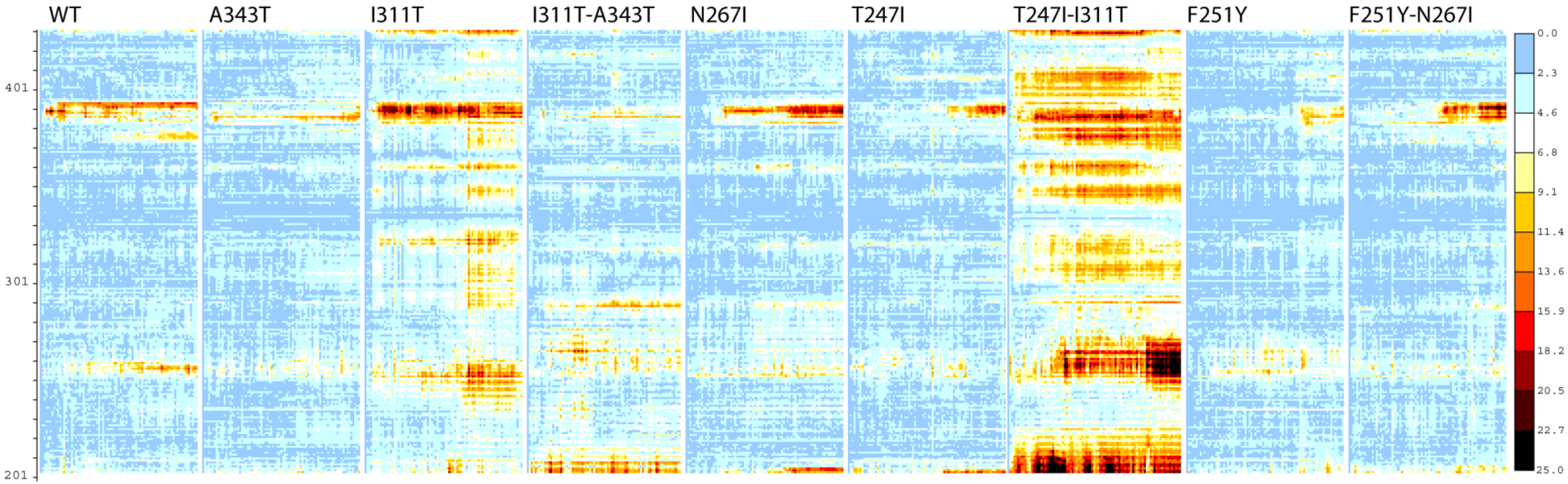

Figure S2. Docked conformation of ATP in the cytoplasmic domain of AgrC. The backbone structure of the WT domain is shown in orange and the ATP molecule is shown in all-atom space-filling representation. Standard colour coding was used for all atoms (C-grey; N-blue; P-orange; O-red; H-white) and hydrogens are not shown; T343 (red arrow) is shown in all-atom representation. Semi-rigid docking assessment was performed using Swiss-Dock (8) and the top ranking binding site was found in agreement with flexible docking using Autodock Vina (9). Molecular visualisation was done in UCSF Chimera (10).

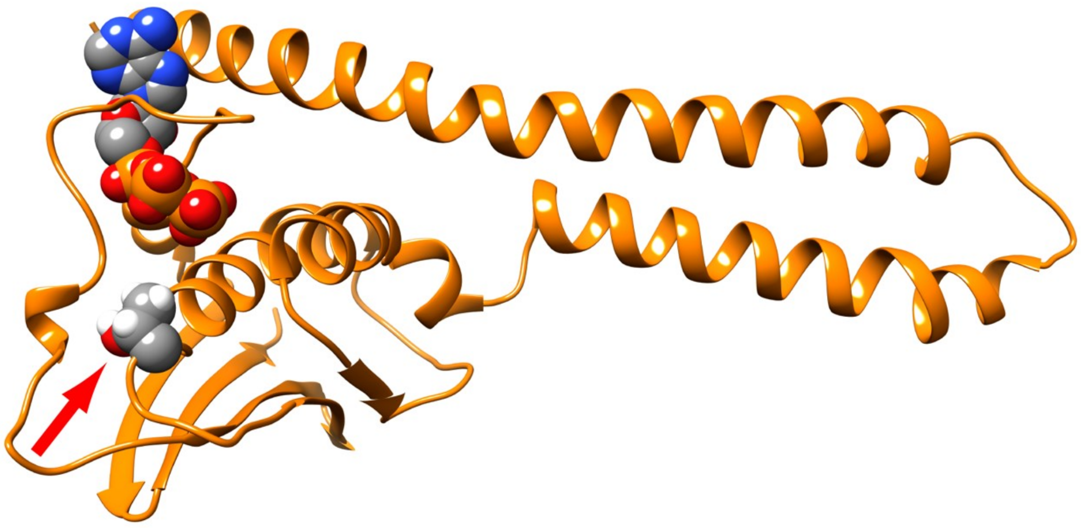

Supplementary references

1. Kreiswirth BN, Lofdahl S, Betley MJ, O'Reilly M, Schlievert PM, Bergdoll MS, Novick RP. 1983. The toxic shock syndrome exotoxin structural gene is not detectably transmitted by a prophage. Nature 305:709-12.
2. Jensen RO, Winzer K, Clarke SR, Chan WC, Williams P. 2008. Differential recognition of Staphylococcus aureus quorum-sensing signals depends on both extracellular loops 1 and 2 of the transmembrane sensor AgrC. J Mol Biol 381:300-9.
3. Lei MG, Cue D, Alba J, Junecko J, Graham JW, Lee CY. 2012. A single copy integration vector that integrates at an engineered site on the Staphylococcus aureus chromosome. BMC research notes 5:5.
4. Otokunefor K, Sloan T, Kearns AM, James R. 2012. Molecular characterization and panton-valentine leucocidin typing of community-acquired methicillin-sensitive Staphylococcus aureus clinical isolates. J Clin Microbiol 50:3069-72.
5. Laabei M, Recker M, Rudkin JK, Aldeljawi M, Gulay Z, Sloan TJ, Williams P, Endres JL, Bayles KW, Fey PD, Yajjala VK, Widhelm T, Hawkins E, Lewis K, Parfett S, Scowen L, Peacock SJ, Holden M, Wilson D, Read TD, van den Elsen J, Priest NK, Feil EJ, Hurst LD, Josefsson E, Massey RC. 2014. Predicting the virulence of MRSA from its genome sequence. Genome Res 24:839-49.
6. Li M, Du X, Villaruz AE, Diep BA, Wang D, Song Y, Tian Y, Hu J, Yu F, Lu Y, Otto M. 2012. MRSA epidemic linked to a quickly spreading colonization and virulence determinant. Nat Med 18:816-9.
7. Humphrey W, Dalke A, Schulten K. 1996. VMD: visual molecular dynamics. Journal of molecular graphics 14:33-8, 27-8.
8. Grosdidier A, Zoete V, Michielin O. 2011. SwissDock, a protein-small molecule docking web service based on EADock DSS. Nucleic acids research 39:W270-7.
9. Trott O, Olson AJ. 2010. AutoDock Vina: improving the speed and accuracy of docking with a new scoring function, efficient optimization, and multithreading. Journal of computational chemistry 31:455-61.
10. Pettersen EF, Goddard TD, Huang CC, Couch GS, Greenblatt DM, Meng EC, Ferrin TE. 2004. UCSF Chimera--a visualization system for exploratory research and analysis. Journal of computational chemistry 25:1605-12.
